# Periodontal status of students living with disability in Amhara region, Ethiopia: a cross-sectional study

**DOI:** 10.1186/s12903-022-02377-x

**Published:** 2022-08-11

**Authors:** Amare Teshome Tefera, Biruk Girma, Aynishet Adane, Abebe Muche, Tadesse Awoke Ayele, Kefyalew Ayalew Getahun, Zelallem Aniley, Semira Ali, Simegnew Handebo

**Affiliations:** 1 Department of Dentistry, School of Medicine, College of Medicine and Health Sciences, University of Gondar, P.O.Box 196, Gondar, Ethiopia; 2 Department of Internal Medicine, School of Medicine, College of Medicine and Health Sciences, University of Gondar, Gondar, Ethiopia; 3 Department of Anatomy, School of Medicine, College of Medicine and Health Sciences, University of Gondar, Gondar, Ethiopia; 4 4Department of Biostatics and Epidemiology, Institute of Public Health, College of Medicine and Health Sciences, University of Gondar, Gondar, Ethiopia; 5 Department of Pharmacology, School of Pharmacy, College of Medicine and Health Sciences, University of Gondar, Gondar, Ethiopia; 6 Department of Special Need and Inclusive Education, College of Education, University of Gondar, Gondar, Ethiopia; 7 School of Public Health, St.Paul’s Hospital Millennium Medica College, Addis Ababa, Ethiopia

**Keywords:** Periodontal disease, Special need, Disability, Oral health status, CPI

## Abstract

**Background:**

Periodontal disease is the most common oral health problem among individuals living with disabilities. Any physical impairment and/or mental handicap can compromise the capability to perform oral health care. Individuals with poor oral hygiene practice were prone to dental caries, periodontal disease, and upper respiratory tract infections. Despite the high prevalence of disabled people in Ethiopia, data are scarce about their periodontal status. The aim of this study was to determine the prevalence and determinant factors of periodontal disease among students living with disability in the Amhara region.

**Methods:**

A **s**chool-based cross-sectional study was done on eight special needs schools in Amhara regional state from November 30, 2020, to April 10, 2021. A simple random sampling technique using a computer random generator was employed to recruit the study participants. The participants were interviewed for sociodemographic characteristics, oral hygiene practice, type of disability, and medical condition through a pre-tested semi-structured questionnaire. The periodontal status of the participants was evaluated using the community periodontal index (CPI). Data entry was done using the Epi-data and analyzed using SPSS 26. Binary logistic regression analysis was used to identify the predictors of periodontal disease at a 5% level of significance.

**Results:**

A total of 443 study participants were involved with a mean age of 15.84 ± 3.882. Among these, 27.5% (95%CI 23.4–32.0) had a periodontal pocket depth of ≥ 4 mm, and 56.7% had bleeding on probing. The prevalence of periodontal disease was higher in participants with poor oral health status (52.2%), dental caries (34.8%), class-2 malocclusion (46.1%), and low monthly income (30.4%), visually impaired (30%), and mentally disorder (29.9%). Age of above 18 years (AOR = 3.41, 95%CI 1.40, 8.28), low family monthly income (AOR = 2.21; 95%CI 1.22, 4.03), malocclusion (AOR = 1.59, 95%CI 1.01, 2.54), poor oral health status (AOR = 9.41; 95%CI 4.92, 17.98), and dental caries (AOR = 1.85, 95%CI 1.21, 2.82) were independent predictors of periodontal disease.

**Conclusions:**

A substantial amount of disabled school students in the study area had periodontal disease. The study found that there was a statistically significant association between age, family monthly income, malocclusion, oral health status, and dental caries with periodontal disease. The implementation of school oral health programs has a great benefit for the oral health status of disabled school students.

## Background

Disability is any condition of the body or minds (impairment) that makes it more difficult for the person with the condition to do certain activities (activity limitation) and interact with the world around them (participation restrictions) [1]. About 10% of the population in developed countries and 12% in developing countries are living with a disability [2]. In Ethiopia, 17.6% of the population had a disability [3]. Globally, the prevalence of disability is increasing due to higher survival rates, an increased aged population, and the increased prevalence of chronic diseases [4–6].

Oral health problems are significant in people with disability that might be associated with their actual disability, other medical conditions, social factors, medications, lack of access to oral health care, and their parents’ neglect of oral health [7]. A reduced mental or physical state, impaired vision, or restricted dexterity can limit individuals' ability to perform adequate oral care [8–10]. Poor oral hygiene practice coupled with low dental health coverage, lack of regular dental check-ups, restorative, and/or surgical treatment in the dental office, leads to an increased prevalence of dental caries, periodontal disease, and respiratory infections [11–14].

Periodontal disease is the most common oral health problem in children with disability, and more than 75% of children with disabilities are unable to obtain needed dental care [15]. Moderately affected, and uninsured individuals were significantly associated with higher odds of having unmet dental needs [16]. Children with a disability had a higher prevalence of periodontal disease than non-disabled children, and there was a significant association between type of disability and periodontal disease [17]. Furthermore, a study done in India on hearing-impaired individuals reported that 45.53% and 2.72% of them had bleeding on probing (BOP) and periodontal pocket depth of 4–5 mm respectively [18].

**Fig. 1:**
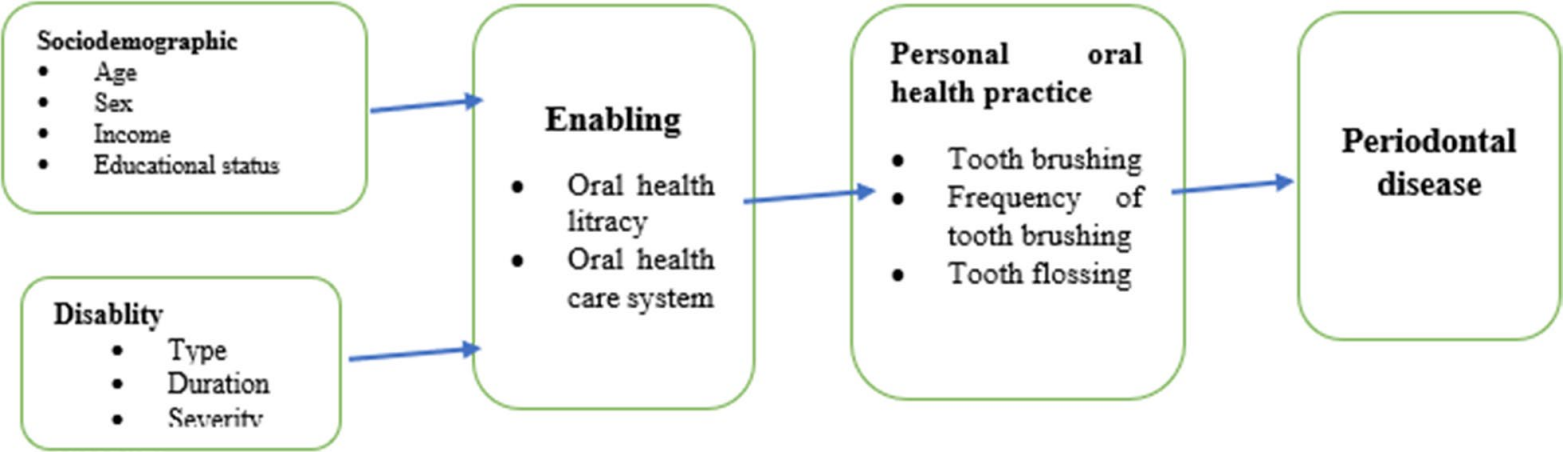
Conceptual frame work

The absence of data regarding the periodontal disease of students with disability in Ethiopia makes it difficult to obtain a conclusion on the prevalence of periodontal disease in this population. Knowing the prevalence of periodontal disease among the disabled population has utmost importance for designing effective preventive and treatment methods. Despite the high prevalence of disabled people in Ethiopia, there is a scarcity of information about their periodontal status. Hence, the present study aimed to assess the periodontal status and associated factors among students living with disability in the Amhara region, Ethiopia (Fig. 1).

## Methods

### Study setting and participants

The study was conducted in special needs schools in the Amhara Regional State of Ethiopia. In Amhara regional state, there are eight special needs schools located in; Gondar, Bahir-Dar, Debre-Markos, and Dessie town. Six hundred ninety-six disabled students are attending special needs schools in the region, and half of them (341) were hearing-impaired students. students who met the following inclusion criteria were included in the study; agreed and signed the consent form (parents gave written consent), attending a special needs school in the region during the data collection period. However, the critically ill, who didn’t give full data due to their disability, uncooperative students, and students living with HIV/AIDS and Diabetic Mellitus were excluded from the study.

### Study design

A school-based cross-sectional study was conducted from November 30, 2020, to April 10, 2021, among students living with disability and attending special needs schools in the Amhara region, Ethiopia. The study design and reporting was in agreement with the ‘strengthening the reporting of observational studies in Epidemiology (STROBE) statement for cross-sectional studies.

### Sample size determination and sampling techniques

The sample size was calculated using the single population proportion formula. Assuming; a 50% prevalence (since no past national data exist), 95% confidence interval, 5% margin of error, and a 15% non-response rate. The final sample size was 443. A simple random sampling technique using a computer random generator was employed to recruit the study participants.

### Measures

#### Disability

A disability is any condition of the body or minds (impairment) that makes it more difficult for the person with the condition to do certain activities (activity limitation) and interacts with the world around them (participation restrictions) [1].

#### Periodontal disease

A student was considered to have periodontal disease if he/she had a periodontal pocket depth of > 3 mm [19].

#### Special need schools

Schools give programs for students who have challenges or disabilities that interfere with learning. Moreover, they provide the support that has not normally provided in general education programs. These schools and programs tailor learning to address each child's unique combination of needs [20].

### Measure collection

A pre-tested structured interview administered questionnaire adapted from the WHO oral health survey tool was implemented [21]. The questionnaire was prepared in English and translated into the local language, Amharic. To check the consistency of the questionnaire, the Amharic version was then translated back to English. Data collected were demographic data, oral health practice, medical condition, type of disability, and presence of oral habits (finger sucking, mouth breathing, etc.). Data regarding the type of disability was obtained from the coordinator of special needs education of each school. The disability condition and demographic data were collected before the intra-oral examination. Data were collected from the selected students with the assistance of their parents or legal guardian. For the intellectually disabled students, parents were interviewed regarding sociodemographic characteristics and other habits of their children.

Data was collected under strict supervision by 8 dental surgeons and two special needs experts. The data collectors received a five-day training on the study's objectives, research ethics, approach to the interviewee, data collection tools and techniques, and confidentiality during study selection of study participants and data collection. The data collectors wrote all answers to the questionnaires. The supervisors (AM, KA) had onsite supervision during the whole data collection period and checked the data daily to ensure its completeness and consistency.

### The dental examination

Four qualified dentists were involved in the clinical examination with the aid of special needs experts and other health professionals. The dentists were trained and calibrated using the WHO oral health survey tool by the investigator. The calibration and standardization of the evaluators were done through a series of training that includes; a theoretical overview, and discuss issues and questions encountered during the examination period. Moreover, a pretest was done on 45 disabled students at Injibara before the actual study to validate the diagnostic criteria. The clinical examination was conducted in the classroom using normal light and students' chairs. Participants suffering from severe physical handicaps and confined to a wheelchair were examined in their wheelchairs. The examinations were conducted with the aid of a mouth mirror, and periodontal probe. The periodontal status, bleeding on probing (BOP) and periodontal pocket depth (PD) around all teeth, and loss of attachment around the six index teeth were evaluated according to the modified community periodontal index criteria (CPI). Moreover, “Guidelines for Periodontal Screening and Management of Children and Adolescents Under 18 Years of Age” was used for evaluation of the periodontal status of under 18 years of age children [22]. Students with a dental emergency were linked to the nearby dental center for treatment. A maximum infection prevention mechanism was implemented by the data collectors to avoid COVID-19 infection.

### Ethical approval and consent

Permission (Ref. No: V/P/RCS/05/541/2020) was obtained from the institutional ethical review board of the University of Gondar, and educational admins of respective zones and heads of schools. The objective of the study was explained to the students, parents, and special needs teachers. Written Informed consent was obtained from all participants and/or their parents/legal guardians for study participation. To ensure confidentiality of data, study subjects were identified using codes and unauthorized persons would not access the collected data.

### Statistical analysis

Each questionnaire was evaluated for completeness and entered into Epi-data (version 4.6) and then transferred to SPSS 26 for coding, storing, and further analysis. Descriptive statistics of categorical variables were presented in terms of frequency and percentage, and continuous variables were presented in terms of mean and standard deviation (SD). Binary logistic regression analyses were done to determine the relationship between the independent variables and periodontal disease. those variables with a *P* value of ≤ 0.2 in the bivariable analysis were transferred to the multivariable logistic regression model to decrease the confounders. An adjusted odds ratio was determined along with its 95% confidence interval and a significant level of *P* < 0.05 was considered for all analyses.

## Results

### Socio-demographic characteristics

Four hundred forty-three study participants were involved in the study and provided a 100% response rate. The mean age of the subjects was 15.84 ± 3.882 years. About 64.3% of the study participants were within the 13–18 years age range. Two hundred thirty-seven (53.5%) of the study participants were males. Almost three-fourths (69.8%) of the study participants were orthodox Christians. Regarding their educational status, 53.3% of the study participants were in grades 1–4. The family monthly income of the study participants showed nearly 2/3rd (62.1%) of them had a monthly income of less than 2500 Ethiopian Birr. Regarding the type of disability, 33.6% of the participants were hearing impaired and 30.9% had a mental disability (Table 1).

**Table 1:** Sociodemographic characteristics of special need school students in Amhara regional state, Ethiopia, 2021 (n = 443)

Variables	Frequency	Percent (%)
Sex		
Male	237	53.5
Female	206	46.5
Age		
7–12	75	16.9
13–18	285	64.3
19–30	83	18.7
Religion		
Orthodox	309	69.8
Catholic	63	14.2
Muslim	62	14.0
Protestant	9	2.0
Location of the participants		
Gondar	92	20.8
Bahir Dar	144	32.5
Debre Markos	133	30.0
Dessie	74	16.7
Grade level		
1–4	236	53.3
5–8	149	33.6
9–12	58	13.1
Mothers educational status		
No education	257	58.0
Read and write	113	25.5
Primary	21	4.7
Secondary	16	3.6
Diploma and higher	15	3.4
Fathers educational status		
No education	197	44.5
Read and write	142	32.1
Primary	22	5.0
Secondary	26	5.9
Diploma and higher	31	7.0
Monthly income		
≤ 2500 Ethiopian Birr	352	62.1
> 2500 Ethiopian Birr	91	10.6
Disability types		
Visual impairment	130	29.3
Hearing impairment	149	33.6
Mental problem	137	30.9
Physical	27	6.1

### Oral hygiene practices

Of 443 study participants, 76.1% had a habit of tooth brushing and 7.9% had brushed their teeth twice and more. Among the students who brushed their teeth, 82.6% had no family support; 13.2% received some support and 4.2% had received frequent support during their tooth brushing. The physically disabled group received slightly higher support (30.4%) than others did while mentally disabled students received low family support (8.5%). Of all students included in the study, 46.7% had poor oral health status. Mentally disabled students had a high frequency of poor oral hygiene status (66.4%) (Table 2).

**Table 2:** Oral hygiene practices among special needs school students in Amhara region, Ethiopia

Variable	Visual impairment (%)	Hearing impairment (%)	Mental disorder (%)	Physical disability (%)	Overall
Tooth brushing habit					
Yes	99 (76.2%)	120 (80.5%)	94 (68.6%)	24 (88.9%)	337 (76.1%)
No	31 (23.8%)	29 (19.5%)	43 (31.4%)	3 (11.1%)	106 (23.9%)
Frequency of tooth brushing					
Sometimes	63 (48.5%)	62 (41.6%)	47 (34.3%/	13 (48.1%)	185 (41.8%)
Once/day	26 (20.0%)	47 (31.5%)	36 (26.3%)	8 (29.6%)	117 (26.4%)
≥ 2/day	10 (7.7%)	11 (7.4%)	11 (8.0%)	3 (11.1%)	35 (7.9%)
Family support during toothbrush					
Never	86 (86.9%)	88 (74.6%)	86 (91.5%)	16 (69.6%)	276 (82.6%)
Sometimes	7 (7.1%)	24 (20.3%)	7 (7.4%)	6 (26.1%)	44 (13.2%)
Always	6 (6.1%)	6 (5.1%)	1 (1.1%)	1 (4.3%)	14 (4.2%)
Oral hygiene status					
Good	21 (16.2%)	54 (36.2%)	6 (4.4%)	6 (22.2%)	87 (19.6%)
Fair	52 (40.0%)	48 (32.2%)	40 (29.2%)	9 (33.3%)	149 (33.6%)
Poor	57 (43.8%)	47 (31.5%)	91 (66.4%)	12 (44.4%)	207 (46.7%)

### Periodontal status

Of the total disabled students, only 28.7% had a health periodontium. More than half of the study participants had calculus deposition. Periodontal disease was present in 27.5% of the participants (18.5% had shallow pockets and 9% in deep pockets). Moreover, more than half (56.7%) of the study participants had bleeding on probing (BOP). Mentally disabled students were more affected by periodontal disease than others were. Males had a slightly higher prevalence of shallow periodontal pocket (PPD 4–5 mm) than females (52.2% vs. 47.8%). Twenty-three students (57.5%) of 13–18 years and thirteen (32.5%) of 19–30 years had deep periodontal pockets (periodontal pocket depth of ≥ 6 mm) (Table 3).

**Table 3:** Periodontal status of the study participants using the Community periodontal index (CPI) in the Amhara region, Ethiopia, 2020/21

Variables	Healthy gum	Bleeding on probing	Calculus	Periodontal pocket of 4-5 mm	Periodontal pocket ≥ 6 mm
Sex					
Male	67	127 (50.6%)	108 (52.2%)	42 (51.2%)	20 (50.0%)
Female	60	124 (49.4%)	99 (47.8%)	40 (48.8%)	20 (50.0%)
Type of disability					
Visual	37	73 (29.1%)	57 (27.5%)	26 (31.7%)	13 (32.5%)
Hearing	42	65 (25.9%)	47 (22.7%)	24 (29.3%)	10 (25.0%)
Mental	39	95 (37.8%)	91 (44.0%)	26 (31.7%)	14 (35.0%)
Physical	8	18 (7.2%)	12 (5.8%)	6 (7.3%)	3 (7.5%)
Age					
7–12 years	64	23 (9.2%)	40 (19.3%)	6 (7.3%)	4 (10.0%)
13–18 years	27	168 (66.9%)	99 (47.2%)	62 (75.6%)	23 (57.5%)
19–30 years	36	60 (23.9%)	68 (32.9%)	14 (17.1%)	13 (32.5%)
Grade level					
Grade 1–4	68	135 (53.8%)	113 (54.6%)	40 (48.8%)	22 (55.0%)
Grade 5–8	43	78 (31.1%)	61 (29.5%)	28 (34.1%)	13 (32.5%)
Grade 9–12	16	38 (15.1%)	33 (15.9%)	14 (17.1%)	5 (12.5%)
Tooth brushing					
Yes	97	190 (75.7%)	160 (77.3%)	63 (76.8%)	31 (77.5%)
No	30	61 (24.3%)	47 (22.7%)	19 (23.2%)	9 (22.5%)
Monthly family income (Ethiopian birr)					
< 1000	89	149 (70.0%)	141 (79.2%)	45 (47.9%)	30 (75.0%)
1000–2500	23	41 (19.2%)	22 (12.4%)	12 (18.5%)	5 (12.5%)
> 2500 birr	15	23 (10.8%)	15 (8.4%)	8 (12.6%)	5 (12.5%)

### Predictors of periodontal disease

To identify the factors that affect periodontal disease, a univariate chi-square test was done on data collected in the questionnaires such as demographic, oral hygiene practice, other oral health problems (malocclusion, dental caries), and carbohydrate intake habits. The results showed that age (*P* = 0.008), monthly family income (*P* = 0.001), oral health status (*P* = 0.000), dental caries (*P* = 0.015), and malocclusion (*P* = 0.001) significantly affected the periodontal status of disabled students (Table 4).

**Table 4:** Predictors of periodontal disease among special need school students in Amhara region, Ethiopia, 2021

Study variables	Periodontal pocket		*P* value
	Yes	No	
Sex			
Male	62 (26.2%)	175 (73.8%)	0.486
Female	60 (29.1%)	146 (70.9%)	
Age			
7–12 years	10 (13.3%)	65 (86.7%)	0.008**
13–18 years	85 (29.8%)	200 (70.2%)	
19–30 years	27 (32.5%)	56 (67.5%)	
Grade level			
Grade 1–4	62 (26.3%)	174 (73.7%)	0.612
Grade 5–8	41 (27.5%)	108 (72.5%)	
Grade 9–12	19 (32.8%)	39 (67.2%)	
Monthly family income (Ethiopian Birr)			
≤ 2500	107 (30.4%)	245 (69.6%)	0.001**
> 2500	15 (16.5%)	76 (83.5%)	
Carbohydrate intake			
Yes	106 (26.4%)	296 (73.6%)	0.084
No	16 (39.0%)	25 (61.0%)	
Tooth brushing habit			
Yes	94 (27.9%)	243 (72.1%)	0.766
No	28 (26.4%)	78 (73.6%)	
Comorbidity			
Yes	18 (31.6%)	39 (68.4%)	0.465
No	104 (26.9%)	282 (73.1%)	
Medication intake			
Yes	15 (28.8%)	37 (71.2%)	0.808
No	106 (27.2%)	283 (72.8%)	
Oral health status			
Good	0 (0.0%)	87 (100.0%)	0.000**
Fair	14 (9.4%)	135 (90.6%)	
Poor	108 (52.2%)	99 (47.8%)	
Class-2 malocclusion			
Yes	35 (46.1%)	41 (53.9%)	0.000**
No	87 (23.7%)	280 (76.3%)	
Dental caries			
Yes	64 (34.8%)	120 (65.2%)	0.015*
No	58 (22.4%)	201 (77.6%)	
Type of disability			
Visual impairment	39 (30.0%)	91 (70.0%)	0.474
Hearing impairment	34 (22.8%)	115 (77.2%)	
Mental disability	41 (29.9%)	96 (70.1%)	
Physical disability	8 (29.6%)	19 (70.4%)	
Malocclusion			
Yes	63	120	0.001**
No	59	201	

**Very significant association

*Significant difference

Age, family monthly income, carbohydrate intake, oral health status, malocclusion, and dental caries were statistically significant during the bivariate analysis and entered into the multivariate logistic regression model as independent variables for the outcomes of periodontal disease. The multivariate logistic regression analysis showed that age, family monthly income, malocclusion, class-2 malocclusion, oral health status, and dental caries were the risk factors for periodontal disease (shallow and deep pocket). Students above 18 years old were 3.41 folds at risk of having periodontal disease than 7–12 years students (AOR = 3.41(95%CI 1.40, 8.23). Moreover, students from low family income had a high risk of acquiring periodontal infection (AOR = 2.21; 95%CI 1.22, 4.03). Students with a malocclusion were 1.59 times more likely to have periodontal disease than students who had normal occlusion (AOR = 1.59 (95%CI 1.01, 2.54), and the odds of having the periodontal disease were high in class-2 malocclusion students (AOR = 2.39 (1.30, 4.42). Poor oral health status was the major risk factor for periodontal disease among special needs students (AOR = 9.41; 95%CI 4.92, 17.98) (Table 5).

**Table 5:** The multivariable logistic regression analysis to show the association between the independent variables and periodontal disease

Variable	Periodontal disease		AOR
	Yes	No	
Age			
7–12 years	10	65	1
13–18 years	85	200	1.28 (0.74, 2.21)
19–30 years	27	56	3.41 (1.40, 8.28)
Carbohydrate intake			
Yes	106	296	1.613 (0.765, 3.401)
No	16	25	1
Tooth brushing habit			
Yes	85	232	1
No	28	77	1.30 (0.72, 2.37)
Oral health status			
Good	0	87	1
Fair	14	135	2.34 (1.151, 6.780)
Poor	108	99	9.41 (4.92, 17.98)
Class-2 malocclusion			
Yes	35	41	2.39 (1.30, 4.42)
No	87	280	1
Dental caries			
Yes	64	120	1.85 (1.21, 2.82)
No	58	201	
Monthly family income			
≤ 2500 Ethiopian Birr	107	245	2.21 (1.22, 4.03)
> 2500 Ethiopian Birr	15	76	1
Malocclusion			
Yes	63	120	1.59 (1.01, 2.54)
No	59	201	1

## Discussion

The objective of this study was to determine the prevalence of periodontal disease and associated factors among special needs school students in the Amhara Region, Ethiopia. The result of this study showed that more than half of the study participants had periodontal changes. Age, family monthly income, malocclusion, class-2 malocclusion, oral health status, and dental caries were independent risk factors for periodontal disease.

The present study found that 71.3% of the special needs school students had some periodontal changes which is similar to a study done in India where 11% of 5–12 years old children with disabilities attending special schools had healthy periodontium [23]. Our study found that 27.5% of the participants had periodontal disease (a periodontal pocket depth of ≥ 4 mm). Our finding is low compared with a study done in Kuwait (61%) [17], India (49.64%) [24], and India (96.5%) [25]. Moreover, 50% of intellectually disabled individuals in Hyderabad, India had gingivitis [26]. However, our finding is high compared with studies done in Nigeria (7.3%) [18], Taiwan (5.4%) [27], and India (2.72%) [28]. The difference might be due to the socioeconomic difference and because of the use of different methods for diagnosing periodontal disease.

The present study found that more than half of our study participants had bleeding on probing which is consistent with studies done in Nigeria [18] and India [28]. The high prevalence of periodontal disease in disabled schoolchildren might be due to the challenges in oral hygiene practice or lack of proper family support during tooth brushing. In addition, the present study showed a direct relationship between age and periodontal disease. Students aged 18–30 years had a higher risk of having periodontal disease than those under 18 years old students. It might be due to the assumption that older age students can take care of their oral health more than younger age groups and didn’t get parents' support during tooth brushing.

Similar to our findings, intellectually disabled groups had a higher mean plaque index and clinical attachment loss of 4–5 mm [29]. This might be due to the underlying congenital or developmental anomalies as well as the inability to receive adequate personal and professional care to maintain oral health [30].

Our finding found a statistically significant association between oral health status and periodontal disease. students with poor oral health status were at high risk of having periodontal disease participants with good oral health status. Similar results were reported in Kuwait (AOR = 8.5, 95%CI 3.5–20.9) [17]. Furthermore, a systematic review and meta-analysis by Lertpimonchai et al. found that participants with poor oral hygiene status were 5.01 times more at risk of having periodontal disease than those with good oral hygiene status (AOR = 5.01, 95%CI 3.40–7.39) [31]. The poor oral health status in the disabled population might be due to their actual disability, social factors, medications especially for the mentally disordered individuals, and their parent's negligence of oral health [7].

Moreover, the present study found a statistically significant association between malocclusion and periodontal disease among special needs school students. Similarly, Bollen [32] reported that individuals with malocclusion had more severe periodontal disease. Moreover, our study showed a higher odds ratio of having periodontal disease among those who had class-2 malocclusion. This might be due to improperly aligned teeth making plaque removal difficult, and predisposing to gingival inflammation and periodontal destruction.

We also found a significant relationship between dental caries and periodontal disease. Our results are similar to a previous study done by Strauss et al. that reported individuals with caries had a higher prevalence of periodontal disease than those without caries [33]. The 4th National Oral Health Survey of China also reported that patients with dental caries were 1.40 times (95%CI 1.24, 1.56) having periodontal disease than non-carious patients [34]. Available evidence on the co-occurrence of caries and periodontitis is still controversial [33]. Consequently, a positive or a negative association between both diseases is still a matter of debate. For example, early studies have reported positive [35] and negative associations [36] but also a lack of association [37]. Furthermore, socio-behavioral aspects and socio-economic status are also associated with the development of caries and periodontitis [38].

In this study, we found a non-significance association between tooth brushing habits and periodontal disease. This finding differs from a previous study in Thailand that reported a 8.25 folds risk of having periodontal disease among those who had poor tooth brushing habits than those who brush their tooth frequently [39]. This might be due that the majority of the study participants in the current study did not have appropriate tooth brushing habits, and almost none of them received caregiver assistance while brushing their teeth.

Similar to our finding, one–third of intellectually disabled individuals had periodontal disease in Nepal (30.8%) [40]. However, our finding is relatively high compared with a study done in Nigeria (20.0%) [18]. This might be due to the difficulty in maintaining oral hygiene and accessing oral hygiene tools in the visually impaired populations in the study area. Also, our study found an inverse relationship between socioeconomic status and periodontal disease which is similar to a study done in the USA [41]. A study done in India also reported that the periodontal condition of mentally disabled children and adults deteriorates as the family income decreases (AOR = 6.06, 95%CI 2.31–9.34) [25]. One possible explanation for such a difference is that periodontal disease may not be predisposed to prevention through non-behavioral measures. Population strategies to prevent gingivitis and adult periodontitis rely on health education and individual behavior change [42].

### Strengths and limitations of the study

To the best of our knowledge, this is the first study that tried to assess the periodontal status of students living with disability in Ethiopia. Moreover, the study was done by multi-professionals; dental professionals, public health professionals, internists, behavioral science professionals, special needs experts, and other professionals were involved. However, we have faced the following difficulties; first, the study only included students attending special needs schools, and disabled students who do not attend special needs education were excluded. Moreover, the self-reported nature of the oral health behavior of the questionnaire has some limitations for disabled participants.

## Conclusions

A substantial number of students living with disability in the study area had periodontal disease. The study found that age, family monthly income, malocclusion, oral health status, and dental caries were independent predictors of periodontal disease. We recommend the implementation of oral health education for parents and primary school teachers on oral hygiene practice, and the need for regular dental visits for individuals living with a disability.

## Data Availability

The data that support the findings of this study are available from Amare Teshome (teferaden@gmail.com) but restrictions apply to the availability of this data, which were used under license for the current study, and so are not publicly available. Data are however available from the authors upon reasonable request and with permission of teferaden@gmail.com.

## Abbreviations

CAL: Clinical attachment loss
BOP: Bleeding on probing
CPI: Community periodontal index
PPD: Periodontal pocket depth
